# The Role and Function of Autobiographical Memory Narratives during the Emotional Processing of Breast Cancer Treatment: An Empirically-Derived Memory Coding System

**DOI:** 10.3390/ijerph20021492

**Published:** 2023-01-13

**Authors:** Maria Luisa Martino, Daniela Lemmo, Joshua Moylan, Caroline Stevenson, Laura Bonalume, Maria Francesca Freda, Jefferson A. Singer

**Affiliations:** 1 Department of Humanities, Federico II University, 80133 Naples, Italy; 2 Department of Psychology, Connecticut College, New London, CT 06107, USA; 3 Department of Clinical Psychology (U.O.S.D), Territorial Healthcare Company, 20873 Brianza, Italy

**Keywords:** autobiographical narratives, memory, breast cancer, adaptation and coping, integration

## Abstract

Breast cancer (BC) in younger age is a critical and potentially traumatic experience that can interrupt the continuity of self-narrative during a crucial phase. In the Narrative Identity framework the translation of memories into autobiographical narratives is an internal and external process that plays a key role in meaning-making, social relationships and self-coherence. The aim of this study is to examine the role and function that autobiographical memory narratives (AMN) play in the process of adaptation to BC medical treatment. Seventeen BC women below 50 years received prompts to provide autobiographical memory narratives at four phases during their treatment (pre-hospitalization-T1-post-surgery-T2-chemo-radio therapy-T3-follow-up-T4). The Emotional Processing Scale (EPS) was also administered. In all, 68 AMN were collected. A three step procedure of data analysis was conducted. The first one, an empirically-derived memory coding manual to analyze key dimensions of AMN was developed: Agency; Emotional Regulation and Interpersonal Relations. Findings show a particular vulnerability in narrative identity faced by BC women during the shift from T1-T3. In the second one, an emotional coping profile for each woman focusing on the shift from T1-T3 was created. For the third step, these profiles were compared with the EPS scores. The final results suggest the capacity of the AMNs to differentiate the women’s emotional adaptation over the course of the BC treatment. Despite the study’s limitations, it supports the use of AMN as clinical device to construct a deeper knowledge and profiling trajectory of how women have internalized and elaborated past encounters with illness and help providers, as well as their prior experience of bodily/psychological health and integrity. This information adds to an understanding of their current efforts at recovery and adaptation. In this way we believe that the recollection of narrative memories, not only at the end of the cancer treatment but also during its process, could help the women to mend the broken continuity of their narrative self, as they seek to maintain a healthy balance of internal resources across their past, present, and projected future.

## 1. Introduction

### 1.1. Breast Cancer Experience in Young Age: The Psychological Impact

The onset of breast cancer (BC) in women under the age of 50 is a critical and potentially traumatic experience that can upset a woman’s life during a crucial phase of her lifespan and the achievement of her personal goals [1,2]. BC is the most common form of cancer in women and the highest incidence is in the 34- to 49-years age group (WHO), with an 87% survival rate. Despite the increasing number of women with BC under the age of 50, the psychological literature on this specific subject, although greatly required by vulnerable and at-risk women, still appears to be limited [3]. In our previous studies, we have shown that breast cancer for younger women increases clinical psychological risks: to feminine-specific concerns linked to the onset within an early phase of lifespan; to a search for resources between individual and relational aspects [1,4,5]. In addition, we demonstrated that psychological symptoms change during the different phases of medical treatment for BC. In particular, the levels of anxiety decrease from the phase of communication of the diagnosis to the chemo/radio therapy, while those of hostility increase. A crucial factor is the capacity of women to emotionally process the traumatic event. This capacity is a strong predictor of psychological symptoms during the phases of medical treatment [6,7,8,9].

The cancer can be interpreted as a stressor due to: the intangible and internal nature of the threat, the uncertainty about the disease outcome, the unpredictable trajectories, and the chronological aspects [10], which present recurrent stressors across different phases of the medical process [6,11]. These characteristics generate an accumulated burden of adversity, which may significantly affect later psychological functioning [12].

From a socio-constructivist and semiotic perspective of the mind [13,14,15,16,17], this event generates a sudden alteration of systems of meaning that support the relationship between the subject and the external world [18,19,20]. There is often a crisis of the sense of continuity of the self-identity [13,21,22]. This crisis is also expressed in an alteration of the temporal perspective that becomes characterized by a sense of fragmentation, a feeling of the suspension of life [22] and an uncertainty about the future [23]. This shift in the relationship to time produces an autobiographical discontinuity and it imposes a narrative urgency in the mind. This intense demand activates the search for a new synthesis of meanings through which to create a space between the pervasiveness of the emotions and the cancer-afflicted self, to give a new order and identifiable structure to these emotions, and ultimately to reorganize the meanings on which the coherence of one’s life story is based [24,25,26,27].

### 1.2. The Role of Narrative and Autobiographical Memory in Critical Experience

Within a Narrative Identity framework [28], autobiographical memory, the focus of the present study, is a particular narration of the self in which the narrator and the protagonist turn out to coincide. Narrative Identity develops and spans across the entire course of life. Individuals reconstruct the personal past, perceive the present, and anticipate the future in terms of an internalized and evolving self-story, which is an integrative narrative of the self that provides a sense of unity and purpose to an individual’s own life [29]. This process leads not only to the organization of events in a coherent way, but also to remembering these events from an integrative perspective. According to Smorti (2011), the relationship between narration and memory is close: personal narratives are comprised of memories, and memories must become narratives to be narrated. Autobiographical narrative is not a simple externalization of autobiographical memory because it implies that life events, maintained in memory, must be reported outside in the form of a story; this action of externalization entails an important transformation process because it provides a narrative structure to memory. Furthermore, the act of storytelling occurs in social relationships which are culturally established, so the shift from the autobiographical memory to the autobiographical narrative is a result from inside to outside (internal and external; cognitive and social) of the individual and they are constructed in the perspective of the present (hic et nunc) that is, in the perspective of the context of remembering and narrating. So, narratives are told to someone and/or to oneself. Consequently, they are even more constrained by the context of narrating and the emotional trigger that activates the process of retrieval and recollection [30,31]. Between memories and the narratives of memories, there is a circular and dynamic relationship of transformation of meanings over time [32,33,34] through the re-emergence of autobiographical material and its structuring into a narrative; people can benefit from cognitive and affective information, but also convert their memories into learning opportunities from experience, extrapolating from memory an integrative meaning that can represent a life lesson or insight [35,36,37].

Studies show that autobiographical narratives memories have a key role to contribute to successful problem solving, coping processes, and pursuing personal goals [38,39]. These kinds of memories serve important directive functions: they inform, guide, motivate, and inspire; they support social relationship and promote self-functions, such as self-continuity (diachronic integration), and they can sustain positive self-regard [40,41].

The process to translate cognitively processed information into “self-defining memories” [42] is our means of connecting specific past experiences to enduring concerns of the general personality system, as expressed through a coherent and continuous sense of narrative identity.

Autobiographical memories, recalled to the level of awareness, reflect the needs of the self. In fact, on the one hand they act as moderators of emotional processes, on the other they play an essential role in protecting and preserving a tolerable self-image. The relevance of some memories and certain objectives per se, with respect to others, derives from the specific responsiveness of the latter with respect to the evolutionary demands that arise in the various stages of life [35,36,37,43]. Specifically, the autobiographical narrative memory, in its constitution as a process, favors: the definition of the self, both in an absolute sense and in relation to others, and the emotional regulation of experiences, contributing, in the latter case, to the maintenance of psychological and physical well-being [34,44,45,46]. Some memories of events lived in one’s existential experience represent essential elements to define who we are; our self, in fact, is shaped by the way in which we manage to remember and reconstruct old experiences, as, by narrating our past, we also narrate about ourselves at the same time [44,45,46,47]. Through the narrative act, people try to weave a common thread between the different memories, which often can appear disconnected, so that they can be configured in a life story and, at the same time, outline a narrative identity; both the life story and the narrative identity contribute, therefore, to a sense of self in the world and in time, with respect to the past, and in the perspective of the future, to find a continuity even in moments of transition [42,44,45,46,47,48,49,50,51,52,53]. 

The construction of a narrative and the attribution of meaning to one’s experiences also play a fundamental role in the process of regulating emotions; many studies, in fact, have investigated the relationship that exists among narration, emotional regulation and autobiographical memory. The narratives, understood as emotionally connoted personal reconstructions, inevitably push people to interface with their emotions, in fact they require adjustment not only during the realization of an experience but also when it is recalled to consciousness and made an object of narration. Researchers have demonstrated that the meaning-making process in response to autobiographical memories is associated with better overall coping and adjustment, while memory content can provide a window into mood disturbances, including depression [35,42].

In this regard, some researchers have highlighted that integrating narratives to achieve a sense of coherence and continuity aids individuals in managing their emotional reactions and provides more control and predictability in their lives [44]. Another aspect closely associated with emotional regulation is the social sharing of emotions. Sharing experiences of affective intensity and strong impact with an interlocutor or more can help to modulate one’s emotions, since talking about one’s own feelings opens one up to the knowledge and awareness of the same elements necessary for the process of regulating emotions [33]. For all of these reasons, it would be of great value from a clinical standpoint to understand the role that memory recollections in the course of primary care consultations as well as diagnostic interviews might play in providing clinicians’ insight into patients’ coping patterns and emotion regulation.

Researchers have demonstrated that the meaning-making process in response to autobiographical memories is associated with better overall coping and adjustment, while memory content can provide a window into mood disturbances, including depression [35,42].

In this regard, some researchers have highlighted that the possibility, on the part of people, of intertwining narratives that convey a sense of coherence and continuity allows them to be able to better manage the emotional reactions associated with events and consequently perceive more control and predictability on one’s life [44]. Another aspect closely associated with emotional regulation is the social sharing of emotions. Sharing experiences of affective intensity and strong impact with an interlocutor or more can help to modulate one’s emotions, since talking about one’s own feelings opens one up to the knowledge and awareness of the same elements necessary for the process of regulating emotions [33]. For all of these reasons, it would be of great value from a clinical standpoint to understand the role that memory recollections in the course of primary care consultations as well as diagnostic interviews might play in providing clinicians’ insight into patients’ coping patterns and emotion regulation.

### 1.3. Autobiographical Memory Narrative and Breast Cancer Experience

Regarding the use of autobiographical memory narrative within the context of breast cancer, some studies attest that when people recall their past in association with life-threatening concerns or conflicts, the disease process can serve as a representative of these events. They have an integrative function because they contain lessons about the self or the world beyond the remembered events [54,55]. This integrated function may indicate that the individual engages in the construction of a life story and uses the past to inform their current experience of illness [56].

Despite the large number of studies in the field of autobiographical memory and the narrative of autobiographical memories, to date, few studies have explored the narrative functioning mediated by autobiographical memory during the course of medical treatment and the role played by them in the process of coping with the experience. Nieto (2019) suggested that intrusive memories of cancer experiences and avoidance related to such events are associated with autobiographical memory issues detected in depressive thinking.

Within the cancer context, studies have mainly focused on the long-survival phase and the construction and quality of an autobiographical memory of the illness experience following medical treatments. In particular, this research investigated the study of deficits in autobiographical memory due to medical treatments and/or psychological symptoms that accompany the traumatic impact of diagnosis and medical treatments.

Although little is known about possible deficits in autobiographical memory function in breast cancer survivors, depression is relatively common among these patients, as well as acute stress syndromes, and post-traumatic stress disorder (PTSD) [57,58,59]. The treatment’s adverse side effects can also induce a poor ability to recollect autobiographical memories [60,61]. In addition, some studies attest that psychopathological factors, such as depression and posttraumatic stress, appear to be more consistently associated with over-generality in autobiographical memory [62]. Moreover, the most anxious patients retrieved fewer emotional details for memories than the controls groups, and had lower self-representation scores than the least anxious patients, who had no deficits in emotional detail retrieval [63].

In one study, memories linked to the breast cancer experience reflected themes of “self-injurious memories”, even after they received successful treatment. Indeed, treatments themselves may bring about notable consequences for quality of life and self-perception. The “injured self” develops in breast cancer patients as illness representation play a crucial role in the consequences, perceptions, emotions, and adherence to treatments. Several daily difficulties could be associated with the Injured Self in terms of psychopathological symptoms and experience of self-fragmentation with negative influences on behaviors [64].

Starting from the aforementioned literature, the aim of this research is the exploration of the role and functions played by narratives of autobiographical memories during the different phases of medical treatment for young women with breast cancer. In particular, through an empirically derived coding system of autobiographical narrative memories, the ultimate goal was to highlight the power of autobiographical memory narratives to differentiate the level/capacity of the women’s emotional coping during the medical treatment. 

## 2. Materials and Methods

### 2.1. Participants and Recruitment 

The research was conducted at the National Cancer Institute Fondazione G. Pascale of Naples, the Italian national referral center for the treatment of neoplastic illnesses. The women who took part in this research were identified from medical reports and assessed for suitability in accordance with the following criteria:

- eligibility criteria were first admission to the hospital before the age of 50 and a diagnosis of infiltrating ductal BC;
- exclusion criteria were metastatic disease (stage IV), neoadjuvant therapy, and psychotherapeutic treatment in progress.

In the first phase of the research, 50 women were recruited at the pre-hospitalization phase. During the study, the total number of women undergoing the four longitudinal phases was 17 (see Table 1), all below 50 years of age (M = 44.47; SD = 3.87). The dropout of women from the first phase of recruitment was due to changes in the hospital structures, a worsening of the cancer condition, a desire on the part of the patients not to continue, and a lack of available time. The meetings took place by means of face-to-face interviews in an ad hoc room of the hospital. The data were collected during the year 2018.

### 2.2. Ethical Approval 

The research was conducted within the framework of the STAR Programme, financially supported by UniNA and Compagnia di San Paolo. The research was co-constructed in collaboration with the hospital’s psychology service and breast unit. The study was approved by the ‘Ethical Committee of The National Cancer Institute Pascale of Naples with managerial decision of N. 36 del 18/01/2018.’ The study respected the American Psychological Association’s Ethical Principles and Code of Conduct as well as the principles of the Declaration of Helsinki. The hospital’s psychology service provided a location and facilities for the monitoring meetings and the treatment of the women who wanted to continue receiving psychotherapeutic support over time. The participants were informed about the study’s aims and procedures and were assured that their participation was voluntary and that their responses would remain anonymous. The women volunteered to participate by providing an informed consent in a written form, with the hospital approving the privacy policy.

### 2.3. Procedure and Longitudinal Recruitment 

Women met during four different phases of the first year of medical treatment. Every medical phase, constituting a turning point of the medical treatment protocols, reflected turning points in the meaning of the woman’s relationship with BC over time and its psychic challenges [54]. 

In Phase I: facing the unknown, the woman is still undergoing diagnostic investigation of a suspected nodularity. 

In Phase II: the impact of the critical valence of the disease, the woman learned about the severity of her pathology (receiving histological examination), has undergone surgery for malignant nodularity, and decides the therapeutic path to be taken.

In Phase III: relationship with a changed body identity, the woman is faced with postoperative chemotherapy or radiotherapy treatments that affect her relationship with her body.

In Phase IV: the construction of a new continuity, the woman returns to the daily routine of life and integrates the maintenance phase, which will last for at least five years. The woman finds herself recovering spaces of autonomy and gradually reducing dependence on the medical institution since she only goes for follow-up.

### 2.4. EPS: Emotional Processing Scale 

EPS-25 is a self-report questionnaire that is designed to identify, quantify, and differentiate the types of EP styles and potential deficits in healthy individuals and those with psychological or physical disorders, as well as to measure the changes in EP as a result of therapy or interventions for physical or psychological disorders, and to assess the contribution of poor EP to the development of psychosomatic and psychological disorders. This scale comprises five subscales, each with five items that are rated on a 10-point (0–9) attitudinal scale: suppression (excessive control of emotional experiences and expressions); signs of unprocessed emotions (intrusive and persistent emotional experiences); unregulated emotions (inability to control one’s emotions); avoidance (avoidance of negative emotional triggers); and impoverished emotional experiences (detached experience of emotions due to poor emotional insights). The total EPS score is obtained by adding the scores of every item completed for the subscale and dividing by the number of items, to give a mean score in a range which goes from 0 to 9. A higher score indicates a poorer EP. The total scores based on a healthy comparison group are as follows: very low (1.1), low (1.7), average (between 2.0 and 5.2), high (5.6), and very high (6.1) [65]. These scores were converted to T scores and scores two deviations above the norm of 50 (70+) were considered high and very high scores, indicating greater dysfunction in emotional processing on each of the subscales.

### 2.5. Narrative Ad Hoc in Deep Interview

We constructed an original ad hoc narrative interview, named the Early Breast Cancer-Processing Trauma Interview (EBC-PTI), to explore young women’s narrative sense-making processes within the BC experience [66] in every phase of their therapeutic path. The same narrative interview during T1, T2, T3, and T4, involved nine open questions that started from the initial request to narrate the illness’s experience from the moment it appeared until the time of the interview. Each question was intended as a narrative prompt that would allow for the recounting of their experience and the opportunity to engage in meaning-making with regard to what they were undergoing. The interview was constructed to activate different forms of narrative discourse and to explore different areas of the experience: area 1, the story of the experience with a specific focus on the actual phase; area 2, attributions about the causes of cancer; area 3, an episodic deepening and examination of relationships with similar experiences—this was the area most likely to activate autobiographical memory narratives; area 4, the specific sense of crisis and change; and area 5, resources.

This study looks specifically at area 3—the prompt regarding of the exploration of episodic memory, and the recollection of autobiographical memories regarding similar past experiences.

The exact prompt for area 3 was: “Could you tell me if you have had other experiences in your life that you consider similar in any way, even if only emotionally, to this one? Could you tell me how you managed them?”

The interview was conducted in an ad hoc room of the hospital; it had an average duration of approximately 40 min and was recorded and then transcribed verbatim. The interview was conducted by two women psychologists who are experts in clinical psychology and narrative methodology. Matching the gender of the interviews and the participants was a conscious choice to promote the women’s open reflection and narration. The researchers were young women; this allowed them to develop an empathic exchange with the patients.

## 3. Data Analysis

### A Step by Step Empirically-Derived Procedure

1. Data Reduction and Translation

In order to examine the specific relationship of the autobiographical memory narratives to the women’s coping capacities across the 4 phases of treatment, the research team extracted these portions of the interviews from the larger interview protocols for each woman. The resulting transcripts contained only the women’s responses to the area 3 prompt for each phase (68 autobiographical memory narratives). Since the autobiographical memory researchers within the research team were English speakers, the first step was to translate the memory narrative excerpts into English. In order to ensure that the transcripts could preserve their meaning after translation and at the same time remain accurate to the original Italian, the American researchers identified two advanced undergraduate students at Connecticut College who were majoring both in Psychology and Italian Studies. After these assistants had translated the memory narrative excerpts to English, the Italian researchers then back-translated a portion of the excerpts into Italian to make sure that the English versions were true to the original Italian. The translations were highly accurate, but any small discrepancies and confusions were clarified through discussion until a full consensus was reached on the veracity of the translations.

2. Development of a Memory Coding Manual System

Although the research team ultimately determined that, with the size of the sample and variation across phases in participants’ responses (both in length and absence of response), a qualitative analysis rather than a quantitative analysis would be most appropriate, the initial procedure was to develop a coding manual that would allow for the assignment of rating values for different variables related to coping and emotional processing.

To develop this manual, the English-speaking research team engaged in a thematic analysis [67] of the 17 participants’ four phases of memory narrative excerpts. The head of the American research team, a faculty member with expertise in qualitative analysis and autobiographical memory (JAS), read through the 17 memory narrative protocols, seeking to identify repetitions and salient coping and affective responses that would yield initial codes. After grouping these codes, three themes emerged: agency; emotion regulation; and interpersonal relations (Table 2). Words and phrases associated with each of these themes can be found in Appendix A. For example, low agency was associated with participants using the word, “helpless”, poor emotion regulation was cued by words like “traumatized”, and low interpersonal support was evidenced by phrases like “I faced it alone”. 

The American researcher then asked one of the undergraduate research assistants (CS) to determine the applicability of these themes by independently coding the memories. After confirmation from the research assistant, these themes were shared with the Italian research group who similarly confirmed the comprehensiveness of these three major themes. On the basis of this consensus, the American team of the faculty member and two research assistants developed The Breast Cancer Survivors’ Autobiographical Memory Narratives Coding Manual (see Appendix A).

Two undergraduate research assistants (one from the original team (CS) and a new second undergraduate assistant (JM)) then used the manual to code and rate all 17 participants’ memory narratives across the four phases of the disease and treatment course. Since the most robust and detailed memory narratives emerged in the first phase (diagnosis) and the third phase (post-surgery and at commencement of chemo and/or radiation treatment), the two assistants paid particularly close attention to the memory narratives from these two phases. Using the coding results, the second research assistant (JM) created 17 emotion coping/processing profiles, synthesizing the agency, emotion regulation, and interpersonal relations findings (Appendix B for the 17 personality profiles). It is important to highlight that these profiles were based solely on the memory narrative data with no other knowledge of the participants and/or their full interview protocols.

## 4. Results

### 4.1. Dividing the Emotion Coping/Profiles

Once the profiles were generated, the faculty member (JAS) and research assistant (JM) divided the 17 participants into two groups on the basis of their profiles—healthier emotional processing and less healthy emotional processing and coping. We made the decision to create the two groups in order to examine how the groups might differ in their scores on the EPS. This would provide us with a pilot approach for assessing the validity of our memory coding system. 

In order to create the two groups, JM conducted a holistic re-reading of each participant’s memory protocols and also reviewed the rating scores on the three dimensions of agency, emotion regulation, and interpersonal support. This review led to the assignment of the 10 participants who showed consistent high levels of struggle across the three dimensions to the less healthy coping group and seven participants to the more healthy coping group. JAS then conducted an independent review of the memory protocols and rating scores; this review confirmed the two groups as being appropriately assigned.

Individuals in the healthier coping group showed more evidence of positive agentic coping (greater confidence in overcoming the disease and its aftermath), more effective emotional regulation (greater optimism and use of more positive coping strategies), and less interpersonal distress (greater connection with others either through receiving/giving support and/or trusting in the medical team and/or their faith). 

### 4.2. Validating the Emotion Coping/Processing Profiles

As the final step in this pilot process, the research team examined the Phase 1 and Phase 3 self-report scores on the five subscales of the EPS for the two groups created from the analysis of the autobiographical memory narrative excerpts. Given the multiple subscale ratings across the phases, the team asked the basic question: in comparing the two groups, what would be the percentage of highly problematic emotional processing scores (defined as two standard deviations above the norm) for the healthier vs. the less healthy emotion coping/processing groups? 

**Figure 1:**
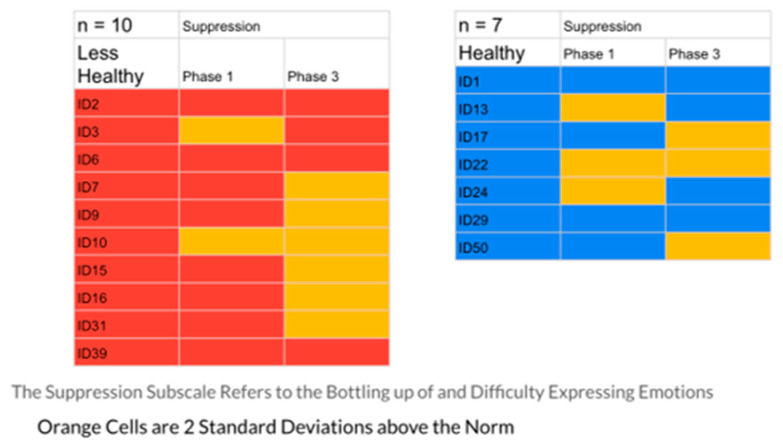
The Suppression Scale EPS: Less Healthy vs. Healthy Coping Groups.

**Figure 2:**
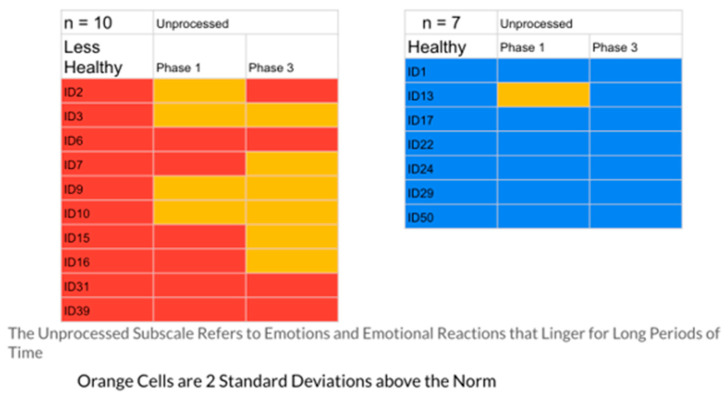
The Unprocessed Scale EPS: Less Healthy vs. Healthy Coping Groups.

**Figure 3:**
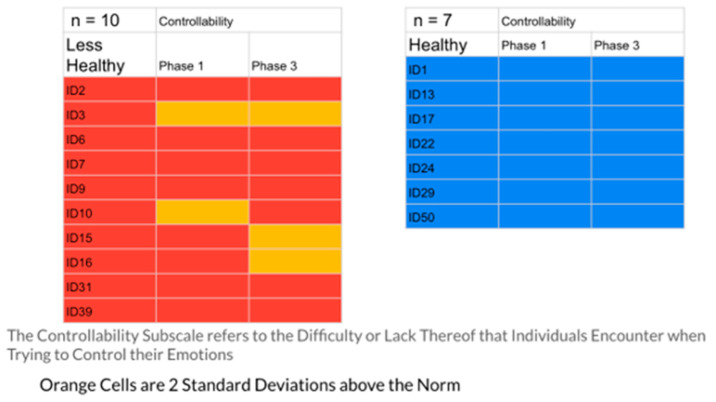
The Controllability Scale EPS: Less Healthy vs. Healthy Coping Groups.

**Figure 4:**
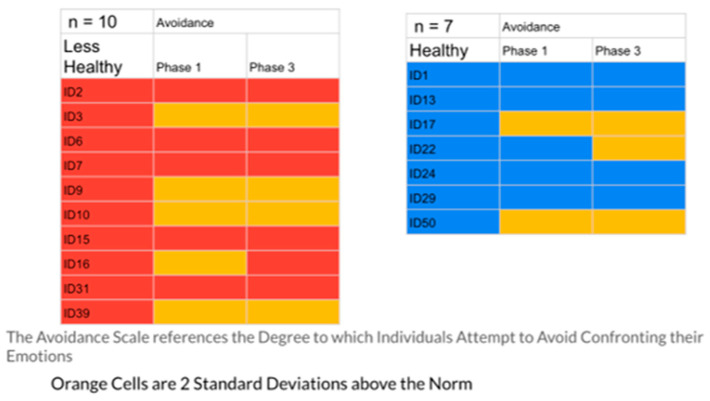
The Avoidance Scale EPS: Less Healthy vs. Healthy Coping Groups.

**Figure 5:**
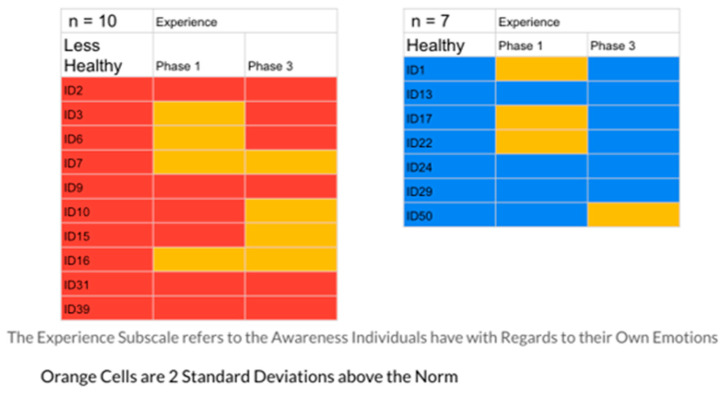
The Experience Scale EPS: Less Healthy vs. Healthy Coping Groups.

The results indicated that the healthier group had 23% of their responses in the problematic range, while the less healthy group had 40%. Figure 1, Figure 2, Figure 3, Figure 4 and Figure 5 display the contrast between the two groups (see Figure 1, Figure 2, Figure 3, Figure 4 and Figure 5). In particular, one can see stark differences in Phase 3 on the suppression and unprocessed emotions subscales. There is also a marked difference summed across the phases for the controllability of emotions. Finally, one can see a much greater awareness of their emotions in Phase 3 for the healthier group. As an additional quantitative analysis, Table 3 depicts the means and standard deviations on the EPS subscales, divided by the healthier and less healthy coping groups. Given the small n’s, we chose to conduct a sign test on the 10 mean comparisons. Nine of the 10 means for the less healthy coping group were higher than the means for the more healthy coping group, indicating greater difficulty in emotional processing, *z =* 2.53, *p =* 0.011 (see Table 3). 

### 4.3. Summary of the Findings

Drawing on previous clinical studies of autobiographical memory narratives’ predictive power [38], the current study examined the heuristic ability of memory narrative excerpts to provide meaningful insight into patients’ personality and coping dynamics. A team of memory researchers isolated and coded 17 breast cancer survivors’ memory narratives taken from extensive interviews over the course of their diagnosis and treatment. With no knowledge of the content of the remainder of the interviews or the specific demographics or details of the 17 survivors, they generated profiles of each survivor by coding their agency, emotional regulation, and interpersonal interactions, as depicted in their autobiographical memory narrative excerpts.

These profiles were then divided into two groups, differentiated by a holistic judgment of the degree of healthy responding to their disease experience. Healthier responses were differentiated by greater expression of positive agency, honest but ultimately optimistic emotional coping, and more adaptive interpersonal functioning (engagement with others and higher degree of trust and faith). To test the validity of this division into two groups, the researchers compared the groups’ self-reported emotional processing scores on the five subscales of the Emotional Processing Scale (EPS). The group defined as less healthy had 40% of their EPS subscale scores at or above 2 standard deviations from the normative scores (indicating less effective emotional processing), while only 23% of the healthier group were at that level. A comparison of means on the subscales for the two groups was also significant. These findings confirmed the effectiveness of the memory coding analysis in differentiating patterns of more and less effective emotional coping styles (see Table 3, Figure 1, Figure 2, Figure 3, Figure 4 and Figure 5)

## 5. Discussion

We find it interesting to focus attention on the presence/absence of some emotional defensive styles in the relationship between EPS and groups of women (healthy and less healthy). Specifically, we refer to the two styles of emotional processing such as: suppression and unprocessed emotion in the less healthy emotion/coping processing group. These defensive styles of emotional processing could represent a block in the access and emotional contact with the current experience of illness that prevents the process of recovering those internalized and integrated autobiographical memories of one’s complex past experiences that can serve as support to the needs of actual self.

On the other hand, the greater awareness and integration of one’s own emotional style of relationship with the experience of illness and treatment in women “more healthy” (this is shown by the total absence of controllability and unprocessed emotions) could indicate that the greater softening of defenses allows access to those elaborated and integrated memories capable of sustaining the current resources and needs of the self. 

The results are further preliminary evidence that autobiographical memory extracts from clinical data can provide valuable insight into individuals’ functioning (agency; emotion regulation; interpersonal relations) and overall coping mechanisms and defenses. This result argues for continued attention to the assessment of memory data from clients in clinical interviews and ongoing clinical interactions. Autobiographical memories offer a window into the overall narrative identity of the individual by revealing the continuity of experience over the life course. A knowledge of how individuals have internalized past encounters with illness and help providers, as well as their prior experience of bodily/psychological health and integrity, may deepen our understanding of their current efforts at recovery and their capacity for resilience.

In conclusion, the analysis of memory data provides an indirect way of ascertaining clients’ psychological health beyond self-report measures of well-being and coping. Although correlated with self-report data, narratives offer a more nuanced and diachronic understanding of coping dynamics—an understanding that helps one to see fluctuations back and forth in coping capacity and strategy.

## 6. Conclusions

The current study is by no means definitive or prescriptive regarding what optimal emotional processing and coping might be in surviving breast cancer and/or serious illness. More modestly, it is a preliminary step in demonstrating the viability and practicality of attending to memory narrative data as a source of meaningful insight in working with survivors over time. Future research would benefit from improved memory narrative measures and continued refinement of techniques of solicitation, extraction, data analysis, and interpretation. 

### Clinical Implications

The current study reinforces the value of autobiographical memory narrative analysis in clinical health, and personality psychology. In particular, the use of narrative memories, collected through narrative interviews during the first phase of illness experience, can offer insight into how these individuals have internalized past encounters with illness and how this internalization reflects their sense of bodily integrity, psychological health, and their relationship to health providers. This knowledge may help providers forecast psychological responses to treatment and aid them in assisting the women in managing and adapting to their breast cancer experience. The clinical health psychology team can plan psychological support consistent with the profiles that emerge from the narrative memories as a means of mitigating some of the coping challenges that are raised over the phases of the cancer treatment. The incorporation of memory narrative assessment into the treatment is another extension of the clinical psychologist’s ability to “observe and listen in depth” to the woman, enhancing holistic treatment that goes beyond the simple mechanics of the diagnosis, surgery, and subsequent adjunctive treatment with radiation and/or chemotherapy.

In addition, clinical engagement with autobiographical memory narratives is also useful at the end of cancer treatment and in the follow-up phase. A knowledge of the survivor’s previous narrative identity structure and themes enables the treatment team to support her efforts to address the discontinuities in her narrative caused by the illness and to rebuild a sense of coherence and sustained meaning, despite an altered sense of self.

### Limitations

There are also several limitations to this preliminary study. First, it was based on a small number of participants, 17 in total, which limited efforts at meaningful inferential statistical analyses. Second, since the original sample was 50 in total, one must be cautious in making generalizations with regard to the 17 participants who persisted across the full study’s four phases. Third, the memory researchers joined this project after the original data had been collected and did not have the opportunity to provide input on the wording of the autobiographical memory narrative prompt. The wording was: “Could you tell me if you have had other experiences in your life that you consider similar in any way, even if only emotionally, to this one? Could you tell me how you managed them?” In future studies, we might suggest using the following wording: “Could you please share a memory from your own life that you might consider similar to what you are undergoing at present? In recalling the memory, please give us some sense of what you were experiencing emotionally at that time, how you felt about yourself, and the role that others in your life played?” This wording, or something similar, might engage the participant to provide a more specific episodic memory with greater detail and imagistic quality. Fourth, the thematic coding of agency, emotional regulation, and interpersonal reactions were inductively developed from the memory narrative protocols of these 17 participants. If the researchers had applied an a priori theoretical lens or had developed the coding manual from a larger sample, a larger number of categories might have emerged along with different emphases in those categories.

## Figures and Tables

**Table 1:** Sociodemographic characteristics of the breast cancer women participated in the study.

Id	Age	Educational Level	Job Position	Marital Status	Number of Children	Age of Children
1	48	Elementary/secondary	Housewife	Married	2	23; 16
2	45	Elementary/secondary	Housewife	Married	2	20; 9
3	39	High school	Housewife	Married	1	7
6	45	High school	Employee	Single	0	0
7	49	Elementary/secondary	Housewife	Married	1	25
9	36	High school	Employee	Married	1	8
10	46	Elementary/secondary	Housewife	Married	2	32; 26
13	47	Elementary/secondary	Freelance	Married	2	21; 11
15	49	Elementary/secondary	Freelance	Married	1	11
16	44	Elementary/secondary	Housewife	Married	3	15; 13; 9
17	41	High school	Employee	Single	2	4; 2
22	42	Elementary/secondary	Employee	Single	3	22; 20; 18
24	48	Elementary/secondary	Housewife	Married	2	19; 14
29	46	High school	Housewife	Single	0	0
31	48	Degree	Employee	Married	2	11; 13
39	44	High school	Employee	Separated	1	22
50	39	High school	Employee	Married	1	9

**Table 2:** Codes of manual system.

Agency	The degree to which the participants felt they could make an active response or have any sense of control in response to their diagnosis, disease process, and treatment.
Emotion Regulation	Encompassed their range of coping responses at each phase of their cancer diagnosis and treatment; these responses included acceptance; distraction/denial; distress/hopelessness; and active positive coping.
Interpersonal Relations	Included accepting support; supporting others; distancing/self-reliance; expressing trust/faith in a greater power, whether God or the medical team; and interpersonal distress caused by negative family and/or other interactions.

**Table 3:** Means and standard deviations for the emotional processing subscales.

Divided by Less Healthy and More Coping Styles Based on Memory Coding				
	Less healthy		More healthy	
	Coping group		Coping group	
	(n = 10)		(n = 7)	
	Phase 1	Phase 3	Phase 1	Phase 3
	Mean (S.D.)	Mean (S.D.)	Mean (S.D.)	Mean (S.D.)
Suppression	4.27 (2.17)	6.20 (2.61)	5.54 (1.94)	4.20 (2.98)
Unprocessed	4.94 (2.79)	5.80 (1.75)	3.83 (2.72)	2.56 (2.00)
Controllability	3.21 (2.46)	4.53 (1.96)	1.37 (1.24)	2.16 (1.62)
Avoidance	4.58 (2.96)	5.20 (2.32)	4.06 (1.26)	4.68 (2.82)
Experience	3.81 (1.62)	3.91 (1.92)	3.33 (1.70)	3.12 (2.21)

## Data Availability

The data presented in this study are available on request from the corresponding author. The data are not publicly available due to the protection of the privacy of the women involved.

## Appendix A

### Breast Cancer Survivors’ Autobiographical Memory Narratives Coding Manual

When asked about a past experience relevant to her current situation, many of the women provided a more summarized account of previous encounters with illness in their own or a family member’s life. They seldom focused on a specific episodic memory, but gave more general impressions regarding this period in their lives. In reviewing the 17 interview protocols that contain 68 responses to the prompts for previous experiences, we discerned three major categories for coding these recollections: The Degree of Agency Experienced; Emotional Expression; and Relationship to Others.

- Degree of Agency Experienced—As past experiences are described, what is the ultimate attitude of the participant toward the outcome and their ability to have an effect or influence on that outcome?
  Since there are four interviews for each participant, the coding sheet should reflect a score for each interview and then a total score. If the participant failed to answer a prompt, then this should be noted and they should be removed from any analyses that aggregate across that prompt. For participants who answered all four interview prompts, their scores can range from a total of 4–12.
  1. Memory Reflects Sense of Passivity and Helplessness in Face of Destiny (Score of 1)
    - Participant uses words like: “helpless, powerless, paralyzed, lost, out of my control”; “how do you fight it?”; “what do you do?”; “I was lost”; “you are missing out on something”; “you don’t have the power to decide about anything”; “it is others who decide for you”; “you just have to stay and wait”; “I don’t feel strong”; “it all went wrong”; “it did not strengthen me”; “I didn’t feel in control of my body”; “it was difficult to recover”; “it is chaos”.
    - Participant indicates: “this is God’s will”; “this is my fate”; “I am a victim of bad luck”; “why me?”; “a sense of resignation”; “bad luck”; “sometimes you wonder why?”; “the disease has put me up against the uncontrollable”.

(Note: ID10 is a special case—she tells a series of connected painful anecdotes of situations in which she is powerless in the face of others’ actions. She does not use exact codable language, but there is no question that the overriding theme is of painful outcomes inflicted on her without her capacity to change these events.)

2. Memory Reflects a Degree of Uncertainty but Commitment to Endure (Score of 2)
    - Participant uses words like: “I will survive”; “it won’t be easy”; “I have to hope for the best”; “I dodged a bullet”; “my faith has given me the strength to survive”; “I am coping with life a little better”; “uou continue on”; “you do the best that you can”.
  3. Memory Reflects Strength and Confidence about Outcome (Score of 3)
    - Participant uses words like: “I will be strong”; “I will go forward”; “I will win this battle”; “I will not let this get me down”; “I can handle this”; “you fight through it”; “I won’t give up”; “I overcame it”; “I dealt with it well”; “I am optimistic”; “I try to be confident”; “I have tried to be strong”; “I mobilized. I tried to do my best” (NOTE—the difference between hoping for the best and doing one’s best); “I was not as strong (as I am now)—I suffered more”; “I am overcoming too”; “I find courage within and keep moving forward”; “I have overcome multiple challenges”; “trust and optimism”; “in every case I have dealt with them”.

II. Expression of Negative Emotion and Efforts at Regulation—*(Two Codes)*

*First, if the participant indicates that they have felt a negative emotion, then this should be scored. Every unique mention (even of the same word) should be scored. We can generate a percentage of negative emotion words out of total words across the four phases. To calculate this, we put the word total across the four phases in the denominator and then put the total number of negative emotion words across the four phases in the numerator. The resulting percentage gives us the amount of negative emotion expressed across the four interviews.*

1. Mention of Emotion: “pain” (in psychological sense); “suffering”; “the loss is felt”; “concern”; “traumatic”; “scares me”; “you will be broken and makes me emotional”; “hit me hard”; “most difficult blow”; “when someone is bereaved, this is how I feel”; “anxiety”; “guilt”; “feel bad”; “shaken”; “makes me feel bad”; “struggle”; “carry this weight inside me”; “fear”; “crying”; “nervous”; “hurt”; “depression”; “afraid”; “terrified”; “feeling of fear”; “feeling like the earth had been pulled out from under me”; “devastating”; ”stressed”; “tough”; “bitter”; “angry”.

*Second, is there any explicit indication of how that negative emotion is being addressed? Is it being communicated to others? Is it being pushed away or denied? Is it serving as a source of constructive guidance forward?*

2. Emotion Regulation: for this coding, you should give one overall score for each interview phase. What is the overall sense of emotion regulation for that particular set of responses to the prompt for that phase? This means that each interview phase should be coded as one of the four categories below. If there seems to be evidence of more than one in the same interview phase, choose the one that seems the most dominant among the competing responses. Over the four interviews, the emotion regulation approach that is most frequently used could be considered the “dominant” approach for that individual.
    - Acceptance/Resignation—“resigned”; “you have to just stay and wait”; “have accepted it but with so many consequences”; “you continue on without remorse, regrets; “without giving you any power to take action but to trust your doctors”.
    - Distraction-Denial—“trying to forget it”; “try to cut yourself off from emotions”; “remove yourself”; “not feel them”; “detach myself from things”; “try not thinking about them”; “trying to distract myself”; “don’t like feeling sorry for myself”; “I don’t remember anything”.
    - Difficulty Coping—“most difficult blow”; “sense of helplessness”; “cannot speak about it”; “reliving emotions”; “traumatized” (different than “traumatic”); “never resigned”; “I cannot do it”; “could not successfully react”; “feel dazed”; “it is more heavy”.
    - Coping—“attending a group”; “coping better”; “you cannot break down”; “optimism, tranquility and trusting your doctors (different from mere acceptance because of the state of optimism and tranquility).

III Interpersonal Relations—Degree of Engagement and Support—*Does the participant mention receiving or offering support to family and/or friends? Does the participant explicitly express distancing or hiding their illness from others? Does the participant express trust and faith in others (e.g., doctors and health providers), including faith in God? In this case, each phase can be coded for as many of these responses to interpersonal relations that seem to be present (this refers to the first five below). After coding all four phases, the interpersonal response that seems most prevalent can be noted as the “dominant overall interpersonal relations response.” For the sixth item, “Family illness and loss,” it should simply be noted if this is mentioned in each phase and what kind (e.g., mother, father, sibling, child, aunt, etc.).*
  1. Receiving Support: “my loved ones have all helped me”.
  2. Giving Support: “I have tried to help whomever in my family”; “worrying and caring for children”; “many daily tasks you have to take care of”; “you fight for your children”; “protection you have for your daughter”; “I have to stay here with my daughters”; “I was responsible for my father”; “I never left him alone”; “you cannot break down and must keep your strength up for your family”; “when you assist someone else it is more difficult”; “always found strength for myself and others”; “I have to go and get my son”.
  3. Distancing and Self-Reliance: “defend my children and not let them see me cry”; “not let others see my suffering”; “didn’t want to talk about things with others”; “I don’t have anybody and I don’t feel like begging for attention”; “I face it alone”; “I am very self-reliant”; “I have earned everything I have”.
  4. Expressing Trust and Faith in Others and/or God: “my faith has given me the strength to survive’; “putting trust in a person, a doctor, a team that follows your case”.
  5. Distress Caused by Negative Family and/or Other Interactions: “as a daughter, I cannot count on her”; “mother’s depression”; “daughter turned her back on her”; “daughter hurting her”; “being taken away from mother”; “not with my husband any more”; “problems with my father”; “hospital wasn’t very helpful”; “doctor had wrong diagnosis”.
  6. Family Illness and Loss: “mother’s illness”; “loss of a newborn”; “I was close to my father and watched him die”; “we were left without a mother”; “brother was murdered”; “miscarriage”; “family unit—whole series of diseases”; “brother was unwell”; “father was in poor health”; “when my mom was unwell, fear of losing her was great”; “lost my father when I was 10”; “sisters had it; one did not make it”; “lost our mother when I was 10”; “lost the baby”; “I was five years old when my mother died”; “mother-in-law had tumor”.

## Appendix B

Coping Profiles
ID 1—Blue (Healthy Coping)
ID1 primarily displays an *agentic* focus that emphasizes moving forward and overcoming adversity (ex. “confident in myself, sure of my strength, of my character, I keep pushing forwards”), *regulates emotions* effectively (ex. “You just have to fight through it and keep moving forward”), and *interpersonal interactions* that dominantly give support (ex. “I have always tried to help whoever in my family was dealing with it”). ID1 undergoes a change from passivity to overcoming across the first three phases (see a change in language from “we will see” to “and so you continue forwards”), in addition to a change in emotional regulation style from resigned to effectively coping (see a change in language from “my turn has arrived” and “sense of resignation” to “confident in myself, sure of my strength, of my character, I keep pushing forwards”). However, there is no displayed change in the dominant style of interpersonal relations: it remains giving support throughout the first three phases.
Overall emphasis on giving to others. Sense of maturity, composed.
ID 2—Red (Less Healthy Coping)
ID2 initially shows us a positive sense of agency in phase 1 (ex. “I am optimistic (with my own cancer)”), but quickly gives way to an all-encompassing *sense of passivity and helplessness* which dominates the remainder of the interview (ex. “I felt like things were going crazy, because this was the only time that I have been truly, truly, truly… how do you say it… powerless to do anything… to change the situation. That was the only time I was truly lost. Indeed, you could say that I was more lost then than I am now!”). They *struggle to regulate their negative emotions* (ex. “how do you fight it? What do you do? What do you know? It’s difficult.”) and *both give help and receive help* (ex. When you have children, you find yourself in situations which you… you wouldn’t want, but you fight for them, you understand?” and “But (my loved ones) have all helped me, my niece, my daughter, they manage to get through to you” respectively). We see no change in their style of emotional regulation; they consistently struggle to deal with negative emotions, and we only see their aforementioned change in sense of agency.
Overall struggles to deal with past and current trauma, but shows initial strength. The experience shifts them to a greater sense of resignation.
ID 3—Red (Less Healthy Coping)
ID 3 shows us a *sense of agency that is dominantly passive* (ex. “you have to just stay and wait… it is others who decide for you, you don’t have the power to decide about anything.”), *dominantly displays a sense of resignation* (ex. you face it but there is not much you can do), and *experiences distress from many interpersonal interactions* (ex. “Of course I didn’t have many points of reference (for how to cope with/address it), she is a weak and sick person and you accept it for what it is. But as a daughter I cannot count on her”). They display changes from phases 1 to 3 in agency and emotional regulation (see changes in language from “how is it possible that yesterday we were all alright, I was fine then, and now this happened” to “I have had to be strong and continue forwards” and from “how is it possible that yesterday we were all alright, I was fine then, and now this happened” to “you face it but there is not much you can do”).
Displays a commitment to endure in the face of adversity, despite its weight. Throughout all phases they display tension about agency, vacillating between a sense of powerlessness and a stoic commitment to endure and manage her adversity.
ID 6—Red (Less Healthy Coping)
ID6 shows an *unchanging dominant sense of passivity and helplessness* (ex. “ It has created so much suffering for me, and therefore (affected) my choices a bit”) and an *emotional regulation* style that starts *resigned* (ex. “I say “death is a part of life, why didn’t I just accept it?” I mean, I have accepted it but with so many consequences”) before moving to *struggling* (ex. “Like I already said there is the time in which I faced my mother’s illness, I cannot speak about it unfortunately”.). They have a *dominant interpersonal relations style* that changes from *distancing* (ex. “I showed myself to be very… let’s say I was “strong”) to *giving* (ex. “I went through the whole process with her from start to finish”).
ID 6 shows an ongoing struggle to leave behind past trauma.
ID 7—Red (Less Healthy Coping)
ID7 displays an unchanging *agentic focus on endurance* (ex. I try to be… confident that I can get through this, I don’t care if they have to take my breasts or if they do chemo to me, but I still want to be close to my children, and that’s all”) and an *unchanging sense of distancing* in regards to *interpersonal relations* (ex. Have tried to be strong and to not let others see my suffering”). They display an *emotional regulation* strategy that shifts from effectively coping to *struggling* (see a change in language from “I confronted (his diagnosis) with so much strength” to “I say this because my father wasn’t a talker and he didn’t say anything, he suffered in silence like I do”).
There is an emphasis on carrying weight alone and hiding pain inside.
ID 9—Red (Less Healthy Coping)
ID9 initially displays an initial *agentic focus on endurance* (ex. “Anyway you deal with it, the fear goes away”), but *gives way to a dominant tendency towards passivity and helplessness* (ex. “I have always been afraid of having to miss out on the (lives of) my two little girls, and it is all like a chain”) They also display an *unchanging struggle to regulate negative emotions* (see “and so there’s just so much fear, the predominant adjective for me is fear”). In addition, ID9 is *primarily a giving person*, and shows a deep commitment to their daughters (ex. “My mother was 40 years old when she died, and I will never do that to (my kids), I will never meet the same end”).
An emphasis on caring for their children coupled with an overwhelming fear to leave them behind.
ID 10—Red (Less Healthy Coping)
ID10 displays an *unchanging passive and helpless nature* (ex. “Eh I find myself today as someone who is nervous, I grind my teeth during the night and… I am not the same as I was before, I have changed”), an *unchanging tendency to struggle with negative emotions* (ex. “This is the strongest pain of my life…”), and an *unchanging dominant interpersonal relations style of interaction distress* (ex. “She does not give me the joy of living, the joy of living with this small grandson of mine, or with her (the granddaughter). Why do you have to be so mean? What have I done to you?”).
Painful anecdotes are used to communicate the trouble that ID10 is having coping with the experience of cancer.
ID 13—Blue (Healthy Coping)
ID13 displays an *agentic focus on endurance* (ex. At “first it was not easy, it was very hard, you do not expect your world to fall apart. I was already going to church (before this), but now I am attending a group and I am coping with life a bit better”), an *unchanging emotional regulation strategy of denial* (ex. “In my personal life… no. I don’t feel like… I don’t want to talk about it”), and an *interpersonal relations style of faith in others and/or God* (ex. “My faith has guided me… my faith has given me the strength to survive.”).
There is an emphasis on faith and a reluctance to confront, speak about, or recall specific painful experiences.
ID 15—Red (Less Healthy Coping)
ID15 possesses a *sense of agency that is primarily passive and helpless* (ex. “So in this case I was angry because I didn’t feel in control of my body”), but it *changes to that from enduring* (ex. “You continue on, without remorse, regrets, without much drama, that perhaps this is not the most serious thing”). They display an *emotional regulation strategy that changes from resignation* (ex. “I experienced it as a thing that just happened”) *to become dominantly struggling* (ex. “It was difficult to recover. In this case I became huge, and I found that even then I could not successfully react”). In addition, they display an interpersonal relations style that is defined by *interaction distress* (ex. “The return to work was incredibly difficult, as you are no longer considered a woman, but a mother.”), but *that began as distancing*.
Their experience is defined by a feeling of lack of control, and a sense that their efforts of denial are ineffective.
ID 16—Red (Less Healthy Coping)
ID16’s *sense of agency is dominantly passive and helpless*, and it does not change (ex. “When the phone rang, I was always filled with anxiety, because I was afraid that it would be the hospital calling with bad news, as it was such a precarious situation”). *Their emotional regulation strategy is dominantly distraction and denial* (ex. “I tried to not… to not think, that is how I am”), but over time she struggles to maintain her efforts at distancing and denial (ex. “Ultimately I feel dazed when I engage with these thoughts, and my mind wanders and it is chaos”). *Her dominant style of interpersonal relations is distancing* (ex. “Let’s say that I was always making myself strong, because my whole family was always busy”).
There is an emphasis on distancing herself from her painful experiences.
ID 17—Blue (Healthy Coping)
ID17 displays a *sense of agency that is initially overcoming* (ex. “I had a bit of a feeling like the earth had been pulled out from beneath my feet when… when he started to get sick, and then when he was gone… but fortunately, he gave the basics (life skills/insight) both to me, and to my sister, so that we could grow up”), and *changes* from this (ex. “I basically was responsible for it, because my mother wasn’t capable of it, and my sister is a little further away from these things, and so I took care of it, I tried to take this all on”) to *passivity and helplessness* (ex. “He did not have this same disease but anyways, essentially, he suffered greatly in his final phase of life, and I experienced (anxiety) very intensely in that period, similar to how I feel in this one”). It crosses the gamut of agency scores. They *struggle regulating negative emotions* (ex. “I never left him alone, I spent every night with him; it was a long and heavy journey”), but they *initially displayed strength in the face of these emotions* (ex. “Eh, as it came, day by day, I centered myself around the things that I had to do for him, I mobilized, I tried to do my best”). *They display an interpersonal regulations style that is dominantly and firmly about giving to others* (ex. “I was very close to my father, throughout the entirety of his illness”).
There is a huge emphasis on taking care of others, and growing in the face of trials and tribulations.
ID 22—Blue (Healthy Coping)
ID22 displays an *agency that is initially passive and helpless in nature* (ex. “There was a somewhat long period in which we found ourselves alone, me and both my brother and my sister”), but over time *becomes more focused on strength and overcoming adversity* (ex. “When the experience is yours personally, you manage to not lose strength”). In addition, she displays a coping strategy that is *initially struggling* (ex. “I have endured bad things but they could be resolved, I was the one who couldn’t understand”) but *shifts to focus on marshaling her strength* (ex. “When you are experiencing it on your own skin you can be stronger”), and an *interpersonal relations style that is dominantly giving* (ex. “But when you assist someone else who is sick it is more difficult”).
Over the four phases, there is a developing emphasis on keeping up her strength and not placing a burden on others. She is hardened by her prior experiences with illness in her family.
ID 24—Blue (Healthy Coping)
ID 24 displays an *agency that shifts from enduring* (ex. “Eh… with patience, you just keep moving forwards, unfortunately I couldn’t do anything about it, it is not the kind of thing that can be cured or operated on”) *to passivity and helplessness* (ex. “In contrast, this has been much more heavy, not so much with the surgery but with radiation, because I experience burning, fatigue, heaviness, everything… it burns, it hurts, and everything goes black”), *but is ultimately dominantly overcoming in nature* (ex. “And now, I am overcoming it too”). In addition, she *ultimately shows that she can effectively cope with negative emotions* (ex. “And now, I am overcoming it too”), but she begins as resigned (ex. “There is nothing to operate on, so there is really nothing that I could do”) and overtime shifts to struggling (ex. “No, no, no. No other experience. This has been so much more… serious”). Her *dominant interpersonal relations style is faith in others or God* (ex. “I went to the day hospital, they did it and then sent me home. I am always going to check how it is, and they say that it’s all fine”).
Over time she learns to cope with the difficult experience, and sees herself overcoming the difficulty.
ID 29—Blue (Healthy Coping)
ID 29 shows an *agentic focus on endurance* (ex. “Your world falls apart a bit, you feel that the years go by, though you always hope that things will go well even if it was not a good experience”) but one that *shifts to an overcoming nature over time* (ex. “I give myself courage, and I go on because that’s the way life is”). She *effectively copes with negative emotions* (ex. “I have always found strength for myself and others”), and *in terms of interpersonal relations, she distances herself from others* (ex. “I don’t have anybody and therefore I don’t feel like begging for attention”).
There is an overwhelming sense of individual strength, she is getting through cancer by herself. There is an emphasis on stoicism.
ID 31—Red (Less Healthy Coping)
ID 31 displays an *agency that shifts from passivity and helplessness* (ex. “In the meantime as I was waiting for the results”) to *overcoming in nature* (ex. “I have overcome multiple challenges in life”), but that is *ultimately dominantly enduring* (ex. “I have overcome so many challenges in my life, but these were manageable challenges overall… graduation, work… These were things in my power. In contrast, this disease has put me up against the uncontrollable, something that possesses you without giving you any power to take action, other than trusting my doctors and being a patient”). She displays an *emotional regulation style that shifts from struggling* (ex. “I lost so much weight because I was working so many shifts, even at night, and I was so thin and so stressed”) to effectively coping (ex. “I have earned all that I have, I have had other challenges and for better or for worse I have always managed to overcome them”), but that *settles as resigned in its nature* in phase 4 (ex. “Something that possesses you without giving you any power to take action”). Finally, her *interpersonal relations style is defined by interaction distress* (ex. “But she said this very calmly, like how I am talking now… she just threw these things in my face”).
There is a struggle here to trust others because of past negative interactions with medical personnel. Although she perceives herself as strong and agentic, the disease creates passivity.
ID 39—Red (Less Healthy Coping)
ID 39 may have blocked painful memories. She alludes to tough times with the death of her mother, but never elaborates. In addition, she says she does not remember anything (ex. “No, fortunately… with my mother I was so young (when she died) that I don’t remember anything. I was 5 years old when she died and I don’t remember anything”).
*An emphasis on denial and distraction with regards to emotional regulation*.
ID 50—Blue (Healthy Coping)
ID 50 displays an *unchanging enduring sense of agency, and an emotional regulation strategy that is dominantly effectively coping* (ex. “Well, with optimism, with the tranquility and optimism that is needed, of course, it is necessary, and then by trusting the doctors… and (those) who are working for you, that is very important, isn’t it?”). However, it *shifts over time to distraction and denial in phase 3*. In addition, she displays an *interpersonal relations style that is dominated by expressing faith* (ex. “Anyways, putting trust in a person, in a doctor, in a team that follows your case. And so (you trust that)…they combine the various things and then, they take the right…the right direction”).
The difficulty of confronting the cancer closes her off in phases 2 and 3, but she does express vulnerability again in phase 4.
Blue Code—healthier emotional processing and coping (seven participants)
Red Code—more problematic emotional processing and coping (10 participants)
